# Non-coding structural variation differentially impacts attention-deficit hyperactivity disorder (ADHD) gene networks in African American vs Caucasian children

**DOI:** 10.1038/s41598-020-71307-0

**Published:** 2020-09-17

**Authors:** Yichuan Liu, Xiao Chang, Huiqi Qu, Joseph Glessner, Lifeng Tian, Dong Li, Haijun Qiu, Patrick M. A. Sleiman, Hakon Hakonarson

**Affiliations:** 1 Center for Applied Genomics, Children’s Hospital of Philadelphia, 3615 Civic Center Blvd, Abramson Building, Philadelphia, PA 19104 USA; 2 Department of Human Genetics, Children’s Hospital of Philadelphia, Philadelphia, PA USA; 3 Division of Human Genetics, Department of Pediatrics, The Perelman School of Medicine, University of Pennsylvania, Philadelphia, PA USA

**Keywords:** Genomics, Mutation, Population genetics, Next-generation sequencing, Neuroscience

## Abstract

Previous studies of attention-deficit hyperactivity disorder (ADHD) have suggested that structural variants (SVs) play an important role but these were mainly studied in subjects of European ancestry and focused on coding regions. In this study, we sought to address the role of SVs in non-European populations and outside of coding regions. To that end, we generated whole genome sequence (WGS) data on 875 individuals, including 205 ADHD cases and 670 non-ADHD controls. The ADHD cases included 116 African Americans (AA) and 89 of European Ancestry (EA) with SVs in comparison with 408 AA and 262 controls, respectively. Multiple SVs and target genes that associated with ADHD from previous studies were identified or replicated, and novel recurrent ADHD-associated SV loci were discovered. We identified clustering of non-coding SVs around neuroactive ligand-receptor interaction pathways, which are involved in neuronal brain function, and highly relevant to ADHD pathogenesis and regulation of gene expression related to specific ADHD phenotypes. There was little overlap (around 6%) in the genes impacted by SVs between AA and EA. These results suggest that SVs within non-coding regions may play an important role in ADHD development and that WGS could be a powerful discovery tool for studying the molecular mechanisms of ADHD

## Introduction

Attention-deficit hyperactivity disorder (ADHD) has a prevalence of ~ 6–8% in children with male patients outnumbering females by almost double^1^. Symptoms persist into adulthood in over two thirds of cases, causing significant life-long impairments^2,3^. In the last decade, multiple studies have attempted to investigate the genetic susceptibility of ADHD, most notably by assessing the enrichment of copy number variations (CNVs)^4^ and single nucleotide variants (SNVs) from genome-wide association studies (GWAS)^5^. However, the current understanding of this complex trait is incomplete and attempts to replicate previous studies have been inconsistent, due in part to the highly heterogenous phenotype of ADHD, as well as other factors, such as complicated molecular mechanisms underlying ADHD networks and limitations of genotyping arrays to study structural variations (SV)^6^. Previous studies also show that the susceptibility of ADHD is more likely to be impacted by biological pathways instead of a particular gene^6–8^, and by structural variations (SVs), such as copy number variations (CNVs), inversions, translocations that may play important roles in the regulation of ADHD gene networks^9,10^. Most of previously published studies have focused on coding regions and have been carried out primarily in patients of European ancestry while intronic and intergenic regions were often omitted from analyses. However, non-coding genomic structural variations and non-coding DNA sequences have been shown to play important roles in many human diseases, including neurodevelopmental diseases such as autism and intellectual disability^11,12^. In this regard, the most recent large GWAS studies on 55,374 individuals, including 20,183 ADHD patients, highlighted that variants in non-coding regions, such as non-coding RNA and intergenic region were significantly associated with ADHD susceptibility^13^. In addition, previous studies have largely focused on European Caucasian populations leaving out studies in African populations and other populations.

To address the limitations of the previous studies of ADHD, in this study we have generated deep whole genome sequencing (WGS) data on 875 individuals, including 205 ADHD patients and 670 non-ADHD controls, in order to explore the impact of SVs, especially SVs within non-coding regions, on the pathogenesis of ADHD. We have also included a significant number of African Americans in the study, including 116 cases and 408 controls, to expand the analysis into another population other than Europeans. The results suggest that SVs within non-coding regions play critical roles in the molecular mechanisms underlying ADHD and that population-specific SVs are present. This information would be useful for future studies of ADHD genetic network regulation and drug development.

## Results

### Exonic/splicing SVs impact structure of genes related to neurodevelopment procedures

**Figure 1:**
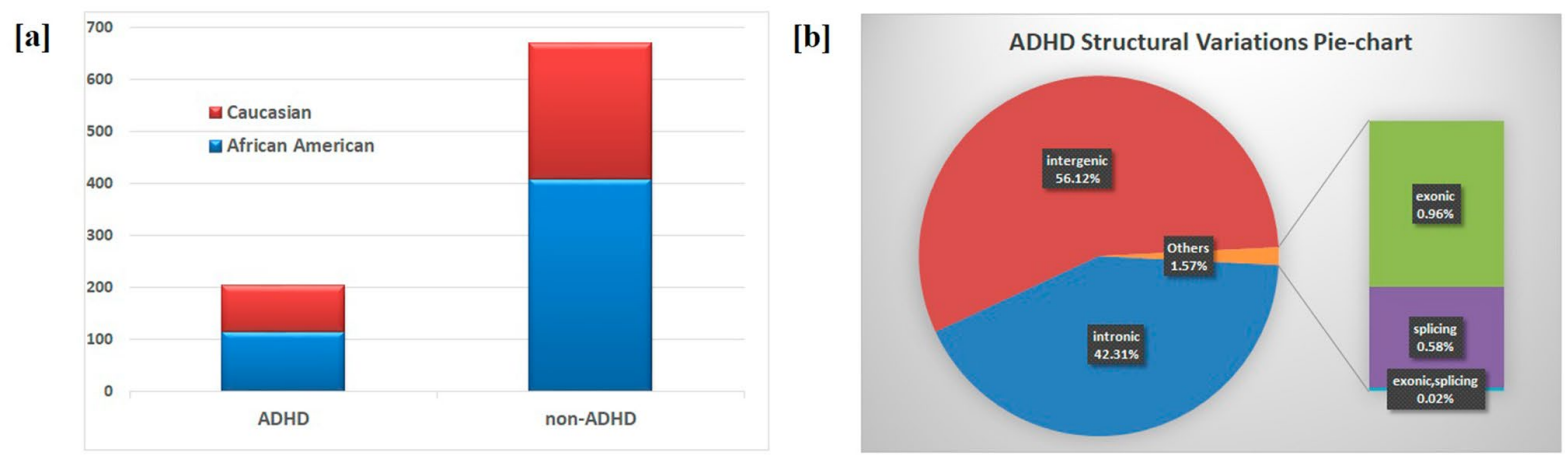
Patient summary and distribution of structural variations (SVs) for ADHD vs control. (**a**) represents the number of ADHD patients and non-ADHD controls with race information; (**b**) distribution of structural variations (SVs) for 206 ADHD patients based on whole genome sequencing (WGS). Intergenic and intronic variations accounted for over 98% of the SVs.

Approximately 160,000 structural variations (SVs) were identified in ADHD patients (Fig. 1b), of those, 0.96% were classified as exonic, 0.59% as splicing, 42.3% as intronic and 56.13% as intergenic. Exonic/splicing usually have more significant impacts since they alter the coding regions and splicing sites directly As expected, they accounted for a small proportion (~ 1.5%) of the total of which 37 were significantly with ADHD threshold 0.05 and 9 with threshold 0.01 (Table 1). In addition to the 37 exonic/splicing SVs that associated with ADHD we identified 451 rare ADHD-associated SVs in AA (Supplementary Table 2a) and 382 in EA (Supplementary Table 2b), 41 SVs are only existed in ADHD cases and were absent from controls (Table 2). A recurrent 320 bp long deletion was identified for three AA ADHD patients at chr5:171723712–171724032, at the splicing site of non-coding RNA *LOC100288254*.

**Table 1:** ADHD-associated Exonic/splicing SVs that passed the statistical threshold 0.01.

Gene_ID	Structure	Type	Num in ADHD	Num in controls	OR	Chi-square p value	Adjusted Chi-square p value	Fisher's Exact p value	Adjusted Fisher's Exact p value	Ethnicity
VPS53	Exonic	Deletion	18	20	3.55	2.57E−04	1	3.79E−04	1	AA
MPEG1	Exonic	Insertion	9	7	4.8	2.61E−03	1	2.82E−03	1	AA
SPATA9	Exonic	Insertion	29	44	2.39	3.84E−03	1	4.01E−03	1	EA
DEPDC1	Exonic	Translocation	6	3	7.33	4.76E−03	1	5.07E−03	1	AA
OR4N4	Splicing	Deletion	70	183	1.87	5.90E−03	1	4.62E−03	1	AA
LOC101927079	Splicing	Deletion	70	183	1.87	5.90E−03	1	4.62E−03	1	AA
LOC100134391	Exonic	Deletion	5	2	9.09	7.19E−03	1	7.22E−03	1	AA
LINC00469	Exonic	Deletion	5	2	9.09	7.19E−03	1	7.22E−03	1	AA
LMLN	Exonic, splicing	Deletion	4	1	14.44	9.99E−03	1	9.79E−03	1	AA

**Table 2:** Rare recurrent exonic/splicing SVs that were only found in ADHD patients.

Gene_ID	Structure	Type	Occurrences in ADHD	Ethnicity
LOC100288254	Splicing	Deletion	3	AA
ANO9	Splicing	Inversion	2	AA
ARHGEF18	Exonic	Insertion	2	EA
BPTF	Exonic	Translocation	2	AA
C20orf27	Splicing	Deletion	2	EA
C20orf27	Exonic	Translocation	2	EA
CASP8	Exonic	Deletion	2	AA
CDHR5	Splicing	Inversion	2	AA
DEAF1	Splicing	Inversion	2	AA
DRD4	Splicing	Inversion	2	AA
EPS8L2	Splicing	Inversion	2	AA
FLG2	Exonic	Deletion	2	EA
FLG-AS1	Exonic	Deletion	2	EA
GCNT4	Exonic	Insertion	2	EA
HMGB3	Exonic	Translocation	2	AA
HRAS	Splicing	Inversion	2	AA
IRF7	Splicing	Inversion	2	AA
KHDC1	Splicing	Translocation	2	AA
LMNTD2	Splicing	Inversion	2	AA
LOC143666	Splicing	Inversion	2	AA
LOC692247	Splicing	Inversion	2	AA
LRRC56	Splicing	Inversion	2	AA
MIR137	Exonic	Insertion	2	EA
MIR210	Splicing	Inversion	2	AA
MIR210HG	Splicing	Inversion	2	AA
NOC2L	Splicing	Duplication	2	EA
PHRF1	Splicing	Inversion	2	AA
PTDSS2	Splicing	Inversion	2	AA
RASSF7	Splicing	Inversion	2	AA
RNH1	Splicing	Inversion	2	AA
SAMD11	Splicing	Duplication	2	EA
SCT	Splicing	Inversion	2	AA
SENP3	Exonic	Deletion	2	EA
SENP3-EIF4A1	Exonic	Deletion	2	EA
SLC35B3	Splicing	Deletion	2	AA
SPART	Exonic	Insertion	2	AA
SV2B	Exonic	Deletion	2	AA
TMEM80	Splicing	Inversion	2	AA
TSACC	Splicing	Deletion	2	AA
WDR72	Exonic	Translocation	2	AA
ZNF585B	Exonic	Translocation	2	EA

### SVs within non-coding regions reveal known and possibly novel ADHD-associated genes

Beside exonic/splicing SVs, we also evaluated association of non-coding SVs in ADHD. The novel intronic SVs are listed in Supplementary Tables 3, 4, 5 for AA, EA, and meta-analysis, respectively. The majority of selected ADHD-associated SV-genes were impacted by SVs within non-coding regions (Table 3), furthermore based on the ADHDgene database^14^, there are no known exonic/splicing SV-genes from previous studies passed the statistic threshold. However, a novel exonic deletion in I*QSEC3* passed the ethnicity meta-analysis (p value = 0.0083, Supplementary Table 6). I*QSEC3* is a neuronal exchange gene related to speech, i.e. childhood apraxia of speech, and down-regulated in autism and schizophrenia^15^.

**Table 3:** Selected SV-associated genes targets based on p value.

Ethnicity	Gene_ID	Structure	Type	OR	Chi-square p value	adjusted Chi-square p value	Fisher's Exact p value	adjusted Fisher's Exact p value	Previous knowledge
AA	RFTN1	Intronic	Translocation	2.88	2.19E−06	0.025	3.52E−06	0.041	Novel
EA	GPD2	Intergenic	Insertion	3.12	1.61E−04	1	2.20E−04	1	Novel
EA	PPEF1	Intronic	Translocation	4.15	1.80E−04	1	2.36E−04	1	Novel
AA	VPS53	Exonic	Deletion	3.55	2.57E−04	1	3.79E−04	1	Novel
AA	NOX4	Intergenic	Insertion	12.95	2.82E−04	1	5.65E−04	1	Novel
AA	DEPDC1	Exonic	Translocation	7.33	4.76E−03	1	5.07E−03	1	Novel
AA	OR4N4	Splicing	Deletion	1.87	5.90E−03	1	4.62E−03	1	Novel
EA	GFOD1	Intronic	Insertion	3.06	7.86E−03	1	6.96E−03	1	Known
meta	IQSEC3	Exonic	Deletion	1.82	8.30E−03	1	6.76E−03	1	Novel
AA	LMLN	Exonic,splicing	Deletion	14.44	9.99E−03	1	9.79E−03	1	Novel
EA	CDH13	Intronic	Deletion	2.14	1.57E−02	1	1.23E−02	1	Known
AA	SLC7A10	Intronic	Insertion	1.81	2.06E−02	1	1.99E−02	1	Known
AA	NTRK2	Intronic	Deletion	1.72	2.33E−02	1	1.88E−02	1	Known
EA	GRM5	Intronic	Insertion	3.58	2.90E−02	1	2.89E−02	1	Known
EA	CLOCK	Intronic	Insertion	1.99	3.57E−02	1	2.84E−02	1	Known
EA	CHRNA3	Intronic	Translocation	2.48	3.70E−02	1	2.90E−02	1	Known
AA	CTNNA2	Intergenic	Deletion	1.63	3.95E−02	1	3.81E−02	1	Known
AA	NRSN1	Intergenic	Duplication	4.53	4.39E−02	1	2.95E−02	1	Known
AA	GRIN2A	Intergenic	Translocation	4.53	4.39E−02	1	2.95E−02	1	Known
AA	HTR1F	Intronic	Deletion	1.59	4.41E−02	1	4.32E−02	1	Known

An example network pathway of SVs within non-coding regions is neuroactive ligand-receptor interaction, a pathway critical in neuronal brain function, known to be highly relevant to ADHD development and regulation of differential gene expression in different ADHD-related brain regions^16,17^. Non-coding SVs such as intronic deletion of *HTR1F*, intronic translocation of *CHRNA3*, intergenic translocation of *GRIN2A*, and intronic insertions of *GRM5*, were found significantly enriched in ADHD patients (Table 4).

**Table 4:** Non-coding SV-genes in neuroactive ligand-receptor interaction pathway.

Gene ID	Name	AA	EA
CHRNA3	Cholinergic receptor, nicotinic, alpha 3 (neuronal)		Intronic translocation p value 0.037
CHRNA4	Cholinergic receptor, nicotinic, alpha 4 (neuronal)	Intronic insertion p value 0.078	
GABRG1	Gamma-aminobutyric acid (GABA) A receptor, gamma 1		Intronic deletion p value 0.061
GRIN2A	Glutamate receptor, ionotropic, N-methyl D-aspartate 2A	Intergenic translocation p value 0.044	
GRM5	Glutamate receptor, metabotropic 5		Intronic insertion p value 0.029
HTR1F	5-Hydroxytryptamine (serotonin) receptor 1F	Intronic deletion p value 0.044	
HTR2C	5-Hydroxytryptamine (serotonin) receptor 2C		Intronic deletion p value 0.073
MC4R	Melanocortin 4 receptor	Intergenic deletion p value 0.073	
OPRM1	Opioid receptor, mu 1		Exonic deletion p value 0.052
OXTR	Oxytocin receptor	Intergenic deletion p value 0.085	

### Structural variations show differences in two ethnicities

**Figure 2:**
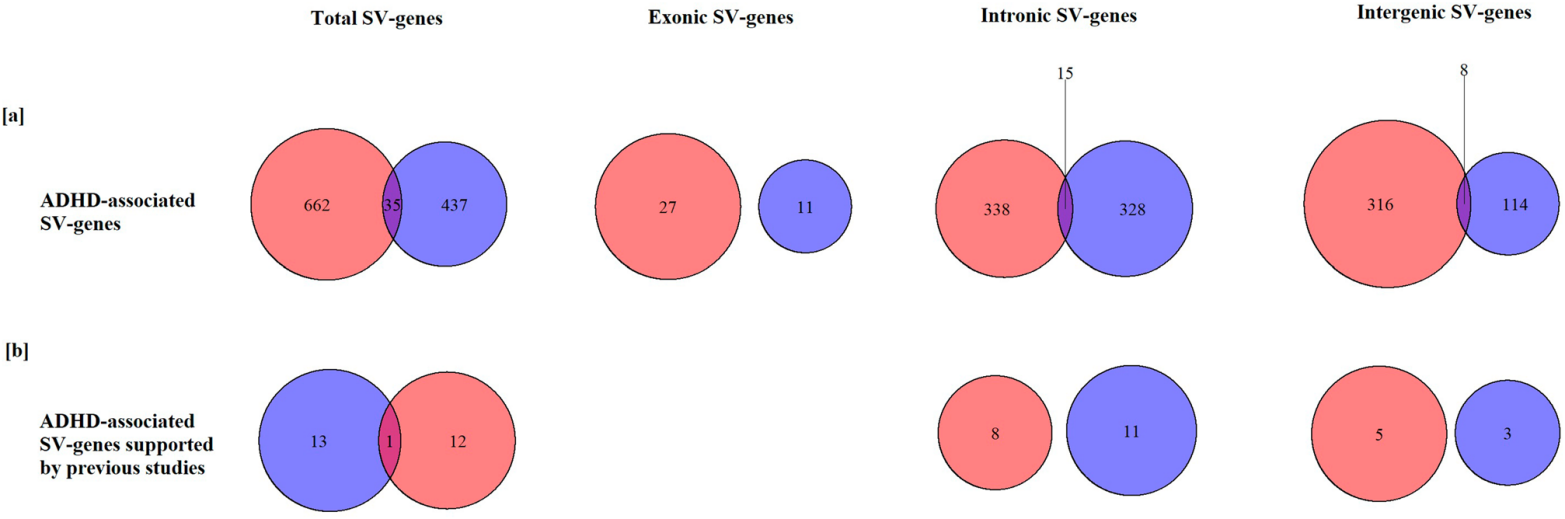
Venn diagram of overlap SV-genes between AA and EA, including all SVs, exonic SVs, intronic SVs, and intergenic SV, respectively. (**a**) SV-genes which significantly associated with ADHD patients (chi-square p value <  = 0.05); (**b**) SV-genes supported by previous ADHD studies and significantly associated with ADHD patients (chi-square p value <  = 0.05).

No obvious differences in SV prevalence types between the AA and EA (Supplementary Fig. 1), however, impacted ADHD-associated SV-genes, which reach statistical significance, are different between two ethnicities (Fig. 2). There were 686 ADHD-associated SV-genes for AA based on statistical tests (Supplementary Table 3), and 439 ADHD-associated SV-genes for EA (Supplementary Table 4). Only 34 genes shared between two ethnicities (Supplementary Table 5), which counted 5%/8% for entire SV-gene set. Meta-analysis identified 234 ADHD-associated SV-genes (Supplementary Table 6), and only four ADHD-associated SV-genes were found in previous literatures (Table 5). Actually, genes in meta-analysis results are still impacted by ethnicities, for example *MYBPC1* has intergenic SVs with p value 0.017 in meta-analysis, and the p value is 0.79 in AA and 0.0032 in EA, in other words, this meta-significant SV-gene passed through meta-analysis because highly ADHD-associated in EA and not significant at all in AA.

**Table 5:** Significant ADHD-associated non-coding SV-genes which have previously ADHD literature support based on meta-analysis.

Gene	Name	Intronic SV significant associated with ADHD	Intergenic SV significant associated with ADHD
MYBPC1	Myosin binding protein C, slow type	–	Yes
CDH23	Cadherin-related 23	Yes	–
KANSL1	KAT8 regulatory NSL complex subunit 1	–	Yes
CDH13	Cadherin 13	Yes	–

## Discussion

Attention deficit hyperactivity disorder (ADHD) is the most common neurobiological disorder in children, with a prevalence of 6–8%^6^. In this study, we identified 37 exonic/splicing SVs, several involving genes that have been previously reported in neurological and mental diseases, such as *VPS53*, which has been previously associated with a neurological conditions and Parkinson disease^18^. Consequently, we identified 40 novel recurrent SV genes associated with ADHD, where the SVs occurred exclusively in ADHD or have frequency less than 0.5% in non-ADHD controls. Those novel recurrent SVs could be also important in ADHD development. For example, we identified a novel 320 bp deletion at splicing sites of non-coding RNA *LOC100288254*, which is a recurrent SV in three independent ADHD patients and only seen in ADHD patients. Additional notable recurrent rare SVs included an exonic insertion of a non-coding RNA, *MIR137*, which has been shown to play a significant role in neural development and neoplastic transformation^19^, splicing inversion in *DRD4* which has previously been implicated in ADHD^20^, and an exonic translocation of *BPTF*, which causes expressive language delay and intellectual disability^21^. *BPTF*, which exonic translocation was identified in two independent individuals, was considered as a candidate gene in neurodevelopmental disorder based on exome pool-seq^22^, and believed to be the cause of syndromic developmental, speech delay, postnatal microcephaly, and dysmorphic features in recent study^21^. We also observed that the non-coding RNA *LINC00461*, which wasone of the 12 significant loci in the study by Demontis et al.^13^, had an intronic insertion in six ADHD patients with chi-square p value = 0.02.

In addition, this study reveals that SVs within non-coding regions may be more critical in ADHD biological networks than they used to believe. One typical example is the neuroactive ligand-receptor interaction pathway, a pathway critical in neuronal brain function, known to be highly relevant to ADHD pathogenesis. In this study, 17 SV genes were found significantly different between ADHD patients and controls, including four genes *CHRNA3, GRM5, HTR1F, GRIN2A* which were supported by previous literature^4,23–25^. All the identified structural variations related to this pathway are either intronic or intergenic. Similar situations were found in other neurodevelopmental pathways, such as MAPK signaling pathway and Axon guidance. Functional role of SVs in non-coding regions in ADHD therefore warrant further investigation. We also show that there is only a small portion of overlap between the two ethnicities of SV impacted genes, and the result was further replicated as we limited the SV-genes to known ADHD genes based on the ADHD gene database^14^. 25 ADHD-associated SV-genes have been previously studied and reported in the literature (Table 6), and only one gene, *AGBL1*, with intergenic SVs shows statistically significant difference in both ethnicities. *AGBL1* was the top locus in the largest ADHD genome-wide meta-analysis done^26^ and mutation in this gene showed significant association with learning performance^27^. Taken together, the results suggest that impacted ADHD-associated genes differ between the two ethnicities, suggesting that ADHD analysis without population information could miss potential disease genes.

**Table 6:** Significant ADHD-associated non-coding SV-genes which have previously ADHD literature support in AA and EA, respectively.

Gene	AA intronic SV significant associated with ADHD	EA intronic SV significant associated with ADHD	AA intergenic SV significant associated with ADHD	EA intergenic SV significant associated with ADHD
AGBL1	–	–	Yes	Yes
CAMK1D	Yes	–	–	–
CDH13	–	Yes	–	–
CDH23	–	Yes	–	–
CHRNA3	–	Yes	–	–
CLOCK	–	Yes	–	–
CTNNA2	–	–	Yes	–
GFOD1	–	Yes	–	–
GPC5	–	Yes	–	–
GRIN2A	–	–	Yes	–
GRM5	–	Yes	–	–
HCN1	Yes	–	–	–
HTR1F	Yes	–	–	–
HTR3B	Yes	–	–	–
ITGAE	–	Yes	–	–
KCTD15	–	–	–	Yes
LINGO2	–	Yes	–	–
MYBPC1	–	–	Yes	Yes
MYO5B	–	Yes	–	–
NCKAP5	–	Yes	–	–
NRSN1	–	–	Yes	–
NTRK2	Yes	–	–	–
SLC6A1	–	–	Yes	–
SLC7A10	Yes	–	–	–
TCERG1L	Yes	–	–	–
TSPAN8	Yes	–	–	–

Small overlap between two ethnicities.

While the majority of those ADHD-associated SVs are located in non-coding regions, the question is how these SVs impact ADHD pathways in the two ethnicity groups: are the pathways different or do the SVs impact same pathways but different gene modules? In order to explore the answers, we further mapped these SVs within non-coding regions into the highly studied ADHD pathways, including neuroactive ligand-receptor interaction pathway. For the ADHD SV-genes which are significantly different in ADHD and non-ADHD controls (p value ≤ 0.05) or have a trend towards significance (p value ≤ 0.1), 10 of them belong to neuroactive ligand-receptor interaction pathway and five genes are AA SV-genes and the left are EA specific SV-genes (Table 4). The results also suggest that SVs, especially SVs within non-coding regions, impact the same gene families but different gene members, such as intronic SVs of *CHRNA3* in AA versus *CHRNA4* in EA, and intronic SVs of *HTR1F* in AA versus *HTR2C* in EA. Based on the enrichment studies for those ADHD-associated pathways, it suggests that the SVs within non-coding regions impact the same ADHD-associated pathways for both ethnicities, but different genes in the same gene families.

In summary, we have conducted a genomic-level study in ADHD patients using whole genome sequencing that takes non-coding genes/regions and ethnicity factors into consideration. The results show that non-coding region SVs and non-coding genes may play a role in the development and progression of ADHD, and WGS may be a powerful tool to explore ADHD molecular mechanisms. Additionally, our study highlights that genomic-level population differences exist between Caucasian and African American patients, especially for non-coding SVs in neuronal genes and that these variants may influence response to specific medications. For the potential evolutional advantages of ADHD in human history^28^, the same ADHD-associated pathways though different genes were involved in the adaption to the environmental selection for survival in the two major human populations. On the other hand, we admit that the current study is limited by the sample size because of the significant expense of WGS. The SVs highlighted by our study warrant further study and confirmation.

## Methods

### Patient selection

The patients were randomly selected from the Philadelphia Neurodevelopmental Cohort (PNC), archived in the biobank of the Center for Applied Genomics (CAG) at the Children's Hospital of Philadelphia (CHOP), with full electronic medical record (EMR). Psychopathology of the cohort was assessed using a computerized, structured interview (GOASSESS)^29^. The diagnosis of ADHD was based on the Diagnostic and Statistical Manual of Mental Disorders-Fourth Edition (DSM-IV) originally, and later confirmed by DSM-V. There were 205 ADHD cases, including 116 African Americans (AA) and 89 European Americans (EA), and 670 controls, including 408 AA and 262 EA (Supplementary Table 1, Fig. 1a).

### Structural variations (SVs) detections

The average coverage for WGS data is 30 ×. The structural variations (SVs), including insertions, deletions, duplications, inversions and translocations, were detected by MANTA Structural Variant Caller developed by Illumina^30^. For quality control, we only included SVs that passed MANTA’s default filters, which required the length of corresponding SVs to be at least 50 bp and rated “PASS” based on MANTA threshold. Passing SVs were stratified into different classes based on their sequence content. Using the hg19 GENCODE reference sequence, if the start and end points of a SV mapped within an exon, it was annotated as “exonic’; if the start and end points of a SV were located within an intronic region and the SV does not spanned an exon, it was annotated as “intronic”; if the SV was located across exon/intron border sites, it was annotated as “splicing”; and the remaining SVs were annotated as “intergenic”.

Exonic, intronic and splicing SVs were annotated with the impacted gene, and intergenic SVs were annotated with their closet gene based on genomic locus. The corresponding annotated gene, either the SVs impacted gene or the closet gene, was named as “SV-gene”. Association of SV-genes in ADHD were calculated for AA and EA independently using Chi-square tests and Fisher’s exact tests. Bonferroni correction was used for correction of multiple testing by the number of tested variations or genes. Significance was set at 0.05 after Bonferroni correction. We only included risk variants in downstream analyses, i.e. SVs that had odd ratios greater than 1. Meta-analysis (combined Chi-square test) was applied when combing two ethnicities together. Pathway analysis is based on DAVID bioinformatics platforms.

### Ethic statement

We confirm that all methods were carried out in accordance with relevant guidelines and regulations and all experimental protocols were approved by the Children’s Hospital of Philadelphia (CHOP). Informed consent was obtained from all subjects or, if subjects are under 18, from a parent and/or legal guardian.

## Supplementary information

Supplementary Information.

Supplementary Table 1.

Supplementary Table 2.

Supplementary Table 3.

Supplementary Table 4.

Supplementary Table 5.

Supplementary Table 6.
