# Supplementary material for: Non-coding structural variation differentially impacts attention-deficit hyperactivity disorder (ADHD) gene networks in African American vs Caucasian children
Source: Sci Rep. 2020 Sep 17;10:15252. doi: 10.1038/s41598-020-71307-0 (PMC7499198; doi:10.1038/s41598-020-71307-0)
Supplement: Supplementary file 1 — Supplementary Information. [file 41598_2020_71307_MOESM1_ESM.docx]

**Non-coding Structural Variation Differentially Impacts Attention-deficit hyperactivity disorder (ADHD) Gene Networks in African American vs Caucasian Children**

Yichuan Liu^1^*, Xiao Chang^1^*, Huiqi Qu^1^, Joseph Glessner^1^, Lifeng Tian^1^, Dong Li^1^, Haijun Qiu^1^, Patrick M.A. Sleiman^1,3#^, Hakon Hakonarson^1,2,3#^

* Those authors contributed equally to this work

# Corresponding author

1. Center for Applied Genomics, Children's Hospital of Philadelphia, Philadelphia, PA, USA

2. Department of Human Genetics, Children's Hospital of Philadelphia, Philadelphia, PA, USA

3. Division of Human Genetics, Department of Pediatrics, The Perelman School of Medicine, University of Pennsylvania, Philadelphia, PA, USA

**Corresponding author:**

Hakon Hakonarson M.D., Ph.D

[hakonarson@email.chop.edu](mailto:hakonarson@email.chop.edu)

Telephone: 267-426-0088

Center for Applied Genomics

3615 Civic Center Blvd

Abramson Building

Philadelphia, PA 19104

Patrick M.A. Sleiman Ph.D

sleimanp@email.chop.edu

Center for Applied Genomics

3615 Civic Center Blvd

Abramson Building

Philadelphia, PA 19104

Running title: Non-coding structural variation impacts ADHD pathways and different between ethnicities

**Supplementary Figure 1.** **Distribution of structural variations (SVs) for ADHD vs control.** [a] distribution of AA; [b] distribution of EA

**
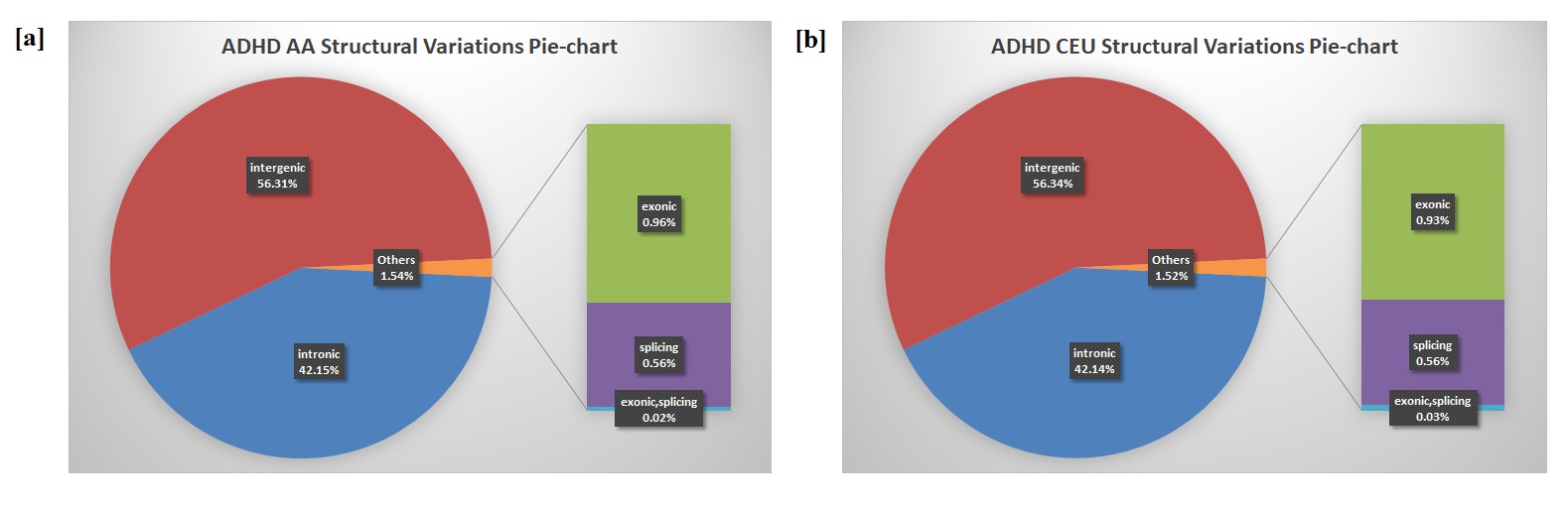
**

**Supplementary Table Legend**

**Supplementary Table 1**. Phenotype for 875 individuals of the study, include ADHD/non-ADHD, ethnicity, and ict9 diagnosis

**Supplementary Table 2**. [a] rare ADHD-associated structural variations (SVs) in African American (AA); [b] rare ADHD-associated structural variations (SVs) in European Ancestry (EA)

**Supplementary Table 3.** ADHD-associated structural variations (SVs) for African American (AA).

**Supplementary Table 4.** ADHD-associated structural variations (SVs) for European Ancestry (EA).

**Supplementary Table 5.** Overlap between ADHD-associated structural variations (SVs) for both ethnicities.

**Supplementary Table 6.** ADHD-associated structural variations (SVs) from meta-analysis based on both ethnicities.
